# Supplementary material for: Hydroxychloroquine-Loaded Chitosan Nanoparticles Induce Anticancer Activity in A549 Lung Cancer Cells: Design, BSA Binding, Molecular Docking, Mechanistic, and Biological Evaluation
Source: Int J Mol Sci. 2023 Sep 14;24(18):14103. doi: 10.3390/ijms241814103 (PMC10531786; doi:10.3390/ijms241814103)
Supplement: Supplementary file 1 [file ijms-24-14103-s001.zip › ijms-2559659-supplementary.pdf]

Quantum chemical parameters

The HOMO and LUMO explain information of the electronic transition of molecules and also indicate the electrophilic and nucleophilic attraction in compounds. Additionally, the chemical reactivity, stability, and hardness of the compounds are explained by the energy gap defined as the difference energy between HOMO and LUMO. A hard molecule is one with a large HOMO-LUMO gap, whereas a soft molecule, high reactivity, is one with the shorter HOMO-LUMO gap. While softness gauges chemical reactivity, hardness gauges molecule stability. From the data of HOMO and LUMO energies, the ionization potential (IP), electron affinity (EA), the energy gap (ΔE) = (IP − EA), absolute electronegativity (χ), electronic chemical potentials (Pi), absolute hardness (η), absolute softness (σ), global softness (S) and global electrophilicity (ω) which have been presented in Table (1). The global reactivity can be defined using Koopman's theorem by the following equations:

$$\chi = \frac{(\mathrm{IP+EA})}{2} \qquad \mathrm{Pi} = -\frac{(\mathrm{IP+EA})}{2} \qquad \eta = \frac{(\mathrm{IP-EA})}{2}$$

$$\sigma = \frac{1}{\eta} \qquad \mathrm{S} = \frac{1}{2\eta} \qquad \omega = \frac{\mu^2}{2\eta}$$

Where, IP = $-E_{\mathrm{HOMO}}$ and EA = $-E_{\mathrm{LUMO}}$
